# Disinfection in and after Fever Cases

**Published:** 1887-05-14

**Authors:** 


### Till the Doctor Comes.

[Inquiries and Communications for this column should be addressed, "Physician," care of the Editor of THE HOSPITAL.]

### XVIII.—DISINFECTION IN AND AFTER FEVER CASES.

In *Enteric Fever* there are numerous small points to be noted. There is in this disease a great tendency to the formation of bed-sores, so that these must be carefully watched for, and treated as before directed. The sufferer lies usually in a low state of muttering delirium, and never asks for anything, so that liquid food, or food, must be given to him constantly, in small quantities, or the mouth will get dry, cracked, and sore, and he will then refuse to take anything. Nasty, brown, foul discharge collects about the teeth and gums, which must be constantly wiped away as before directed. This disease has always accompanying it ulceration of the bowels, so that nothing but liquid, or later semi-liquid food must be given, even after the fever has passed away. If meat or solid food is given too soon, it causes irritation and fresh ulceration over the healed sores, and is a frequent cause of relapse. It is probable that the infection in enteric fever is contained for the most part in the discharges from the bowels. These should be received into a bed-pan containing some disinfecting powder, should immediately be covered with more of the same powder, should be then taken away directly and buried in a deep hole, away from any house, water-course, etc., or they may be more safely burnt immediately.

*After Measures.*—When a case of illness from any infectious disease has terminated, the sick-room and its contents must be thoroughly disinfected and cleansed. Everything that can be destroyed without much loss should be burnt. The different articles and clothing, bedding, etc., that remain should be hung on lines about the room, the chimney, windows, doors, and all crevices should be stopped, and about half a pound of sulphur should be burnt in the room. This is done by putting the sulphur, broken into small pieces, into an iron vessel, supported by the tongs over a pail of water to prevent the risk of fire, and adding some red-hot coals to it. The room must be immediately closed, and left for a few hours. It is necessary to remember that the fumes from burning sulphur are very poisonous. After this, all doors, windows, etc., must be thrown open, and remain so for some days. All paper should then be stripped from the walls and burnt, the ceiling should be whitewashed, the floors, and all paint, walls, furniture, etc., scrubbed with carbolic soap and water, and the chimney carefully swept. Mattresses and things that cannot well be washed should, if possible, be disinfected by heat in a proper chamber or disinfecting oven. There ought to be one of these in every large town, where such articles could be disinfected at a fixed rate. All things that are sent to the wash should be boiled for some time, and some carbolic acid added to the water. The bedsteads, etc., must be washed with a solution of sulphurous acid, care being taken not to touch the brass-work, which would tarnish at once.

No children should be allowed to attend school from a house where there is infectious disease till they can bring a medical certificate that there is no fear of infection. In case of death, there should be no delay in the burial of the body, and plenty of chloride of lime or carbolic powder should be placed in the coffin.

A few words in conclusion as to the disinfectants to be recommended. This must vary a good deal according to the purpose which they have to fulfil.

To disinfect thoroughly an empty room, nothing can compare with the fumes of burning sulphur, used as above directed. When a patient is lying ill in the room, no disinfectant will do the least good, unless used to such an extent as to be dangerous to the patient. The practice of making the room smell horribly with chlorine from chloride of lime is useless and dangerous also, as chlorine is a most irritating gas. Some sanitas sprayed about the room is the only thing we can then advise; it does no good as a disinfectant, but it has a most pleasant, agreeable smell, and refreshes and purifies the room.

As a pure disinfectant nothing is so good as carbolic acid, either as a liquid or powder, for the bed-pan, soiled linen, etc. The great objection to it is its smell. Its use must be advised, but if it is strongly objected to, chloralum, which is without smell, as well as being non-poisonous, may be recommended. Permanganate of potash (Condy's fluid) is good, but will stain very much. Chloride of zinc is also good, but is very caustic, unless much diluted.

---

*To take a Patient's Temperature.*—This is done by an instrument known as a self-registering clinical thermometer. The ordinary temperature of the body is 98.6° Fahrenheit. The index must first be shaken below this, by giving the thermometer a sudden swing or jerk with the bulb downwards. The bulb is then placed in the patient's armpit, and the arm held closely to the side for five minutes by the watch. The thermometer is then removed, and the temperature read off, as the index remains fixed till again shaken. Care must be taken that the bulb touches the skin on each side, and that no clothes intervene whilst it is in the armpit.

